# Role of simple descriptors and applicability domain in predicting change in protein thermostability

**DOI:** 10.1371/journal.pone.0203819

**Published:** 2018-09-07

**Authors:** Kenneth N. McGuinness, Weilan Pan, Robert P. Sheridan, Grant Murphy, Alejandro Crespo

**Affiliations:** 1 Modeling and Informatics, Merck & Co., Inc., Kenilworth, New Jersey, United States of America; 2 Biochemical Engineering and Structure, Merck & Co., Inc., Rahway, New Jersey, United States of America; Russian Academy of Medical Sciences, RUSSIAN FEDERATION

## Abstract

The melting temperature (Tm) of a protein is the temperature at which half of the protein population is in a folded state. Therefore, Tm is a measure of the thermostability of a protein. Increasing the Tm of a protein is a critical goal in biotechnology and biomedicine. However, predicting the change in melting temperature (dTm) due to mutations at a single residue is difficult because it depends on an intricate balance of forces. Existing methods for predicting dTm have had similar levels of success using generally complex models. We find that training a machine learning model with a simple set of easy to calculate physicochemical descriptors describing the local environment of the mutation performed as well as more complicated machine learning models and is 2–6 orders of magnitude faster. Importantly, unlike in most previous publications, we perform a blind prospective test on our simple model by designing 96 variants of a protein not in the training set. Results from retrospective and prospective predictions reveal the limited applicability domain of each model. This study highlights the current deficiencies in the available dTm dataset and is a call to the community to systematically design a larger and more diverse experimental dataset of mutants to prospectively predict dTm with greater certainty.

## Introduction

Protein thermostability is defined by the temperature at which half the protein population is in a folded state, i.e. melting temperature (Tm). Increasing the Tm of a protein is an important goal in biotechnology and biomedicine when designing proteins to remain functional in non-native environments. [1] Improving protein thermostability is expected to lengthen the shelf-life of therapeutics by expanding accessible storage conditions [2], reduce undesired aggregation [3], maintain biocatalytic function of enzymes during harsh bioprocess and manufacturing conditions [4,5], and enhance crystalizability [6] of membrane proteins for structure determination [7]. Protein thermostability is easy to measure, but for a given protein of fixed length, the number of possible single-point mutants could be very large and impractical to explore experimentally. There has been great interest in the development and application of computational methods [8] to help focus and guide the mutant selection process experimentally.

Nevertheless, predicting protein thermostability is difficult because protein folding is expected to be controlled by many weak forces which depend on the environment of the residue being mutated. [9–11] Most computational methods for enhancing thermostability focus on optimizing a small subset of molecular interactions known to impact thermostability such as flexibility, hydration, and patterns of hydrogen bonding, pi-pi and cation stacking, salt-bridges and hydrophobic interactions, rather than predict the impact single-mutations have on dTm itself. These strategies include optimizing surface cavity space [12] and interactions [13], shortening loops [14], enhancing core packing [15–17] and dipole moments [18], increasing the number of disulfide bonds [19], and incorporating non-natural amino acids as stapling agents [20]. Still there is great need for developing efficient and accurate computational methods for predicting dTm.

Efforts have been made to describe the dTm of water-soluble proteins using first principles. Differences in structural fluctuations of molecular dynamics (MD) trajectories have been shown to correlate with changes in thermostability. [21,22] However, MD is not tractable for mutant triage. Additionally, calculated ddG of unfolding has been used to predict the classification of change in thermostability as a result of single point mutations. [23] As a first approximation (e.g. assuming the mutation does not disrupt two-state unfolding, cause major structure perturbation, and change enthalpy of interactions), ddG is proportional to dTm. [24] However, correlation of ddG and dTm is mutation and protein [25] dependent. Additionally, dTm is intricately more challenging to predict than ddG do to the temperature dependence of solvent and amino acid interactions during the unfolding process. [26] Thus, dTm is difficult to reliably estimate from calculated ddG without complete knowledge of how free energy of unfolding varies with temperature.

A promising method for predicting dTm is the use of Quantitative Structure Property Relationship (QSPR) models. QSPR models have been trained on single-point mutation datasets using knowledge-based information, statistical potentials and protein properties as descriptors. [27–31] These models show some amount of predictivity: 5.1–3.0°C mean absolute error (MAE), 0.54–0.27 linear correlation (r^2^), and 75–85% accuracy (Q) (Table 1). Saraboji et al. [28] used the average dTm of each type of substitution (e.g. F->A). While this has the virtue of simplicity, it does not take into account the environment of the residue being mutated and depends on a small set of experiments. Topham et al. [27] used environment-specific substitution probabilities for the mutant secondary structure, solvent accessibility, and hydrogen bonding patterns, derived from structural alignments, to correlate the stability and dTm of a small number of mutants. Machine learning models trained on different classes of descriptors have been used to predict dTm. Masso et al. [29] developed AutoMute a software that uses a four-body, knowledge based, statistical contact potential to predict dTm by calculating residue environment scores from local environment identity and change with respect to wild-type and mutant amino acid secondary structure, buriedness, and closest contact descriptors. Pucci et al. [31], in addition to deriving statistical potentials from distance, torsion, and solvent accessibility descriptors within HotMusic, incorporated temperature dependent potentials extracted from mesostable and thermostable protein subsets to predict dTm. Jia et al. [30] found that the calculated ddG of unfolding from Rosetta was the most important descriptor for predicting dTm when training models using the change in amino acid properties, secondary structure, solvent accessibility and calculated ddG of unfolding.

**Table 1:** Comparison of existing dTm prediction methods dataset statistics Q (accuracy), MAE (mean absolute error), and r^2^ (pearson correlation).

Method	N	^b^Min./Mut.	r^2^	MAE (°C)	Q
Topham et al. [27]	28	NA	0.54	5.1	0.75
Saraboji [28]	1791	0.01	0.32	3.3	0.85
Masso et al. [29]	1749	0.50^c^	0.37	3.0 **[34]**^a^	0.83
Jia et al. [30]	799	101	0.27	NA	0.85
Pucci et al. [31]	1626	1^d^	0.37	3.4 **[34]**^a^	NA
This paper–AL	1626	9*10^−3^	0.33	3.4	0.82
This paper–Cart_tiE_ALG	1626	21	0.46	2.8	0.84

^a^Statistics presented as root mean squared deviation and converted to MAE using ~0.8 factor

^b^Calculation time (Minutes per mutant)

^c^http://binf2.gmu.edu/automute/AUTO-MUTE_Stability_dTm.html

^d^https://soft.dezyme.com/

These QSPR models, to our knowledge, have not been prospectively tested in a protein design campaign, the true test of model performance. Choice of model to be used in protein design campaigns is hindered by the lack of assessment of model scope or applicability domain (AD) within the literature. Assessment of AD is recommended for proper QSRP model validation [32] and critical for evaluating the uncertainty of a dTm prediction for each mutation. Training set identity defines model AD, however the relationship between predicted mutant and training set has not been studied. It is unknown if a model can reliably predict mutant types not found or poorly represented in the training set, or mutations in proteins with low homology (e.g. soluble and membrane protein) or different unfolding kinetics (i.e. two- vs multi-state unfolding) than those in the training set. Complicating assessment of all available models, top models [31,33] have restrictions on the design of protein structures not found in the PDB.

The success of training QSPR models using calculated ddG, amino acid and structure descriptors [30] inspired us to explore how robust these methods are and determine if there are other amino acid, structure, or energy descriptors that can be used to help predict dTm. In this paper, we explore the predictive performance of protein descriptors that are easily calculable from only a 3D PDB structure file. We expand previous work by making models that include amino acid, local and global structure, and the functional terms of calculated ddG of unfolding, from a diverse set of molecular mechanics packages, as descriptors and investigate their importance on predicting change in protein thermostability. We test the hypothesis that using descriptors relevant to the environment of the mutant within the 3D structure of the protein will enhance dTm prediction, recover known factors that impact thermostability, and help realize alternative protein properties that may easily be calculated and useful to consider when optimizing thermostability.

The objective of this paper is to broaden previous research using calculated ddG as a predictor of dTm and answer three main questions that are outstanding in the literature: 1) Does retraining ddG functionals to predict dTm improve performance over using calculated ddG directly from molecular mechanics packages?, 2) Is there a set of simple amino acid and structural descriptors that are easily calculated from only a single crystal structure that perform as well as more complicated models?, and 3) Do models trained with energy and non-energy descriptors perform better than either alone?

**Fig 1:**
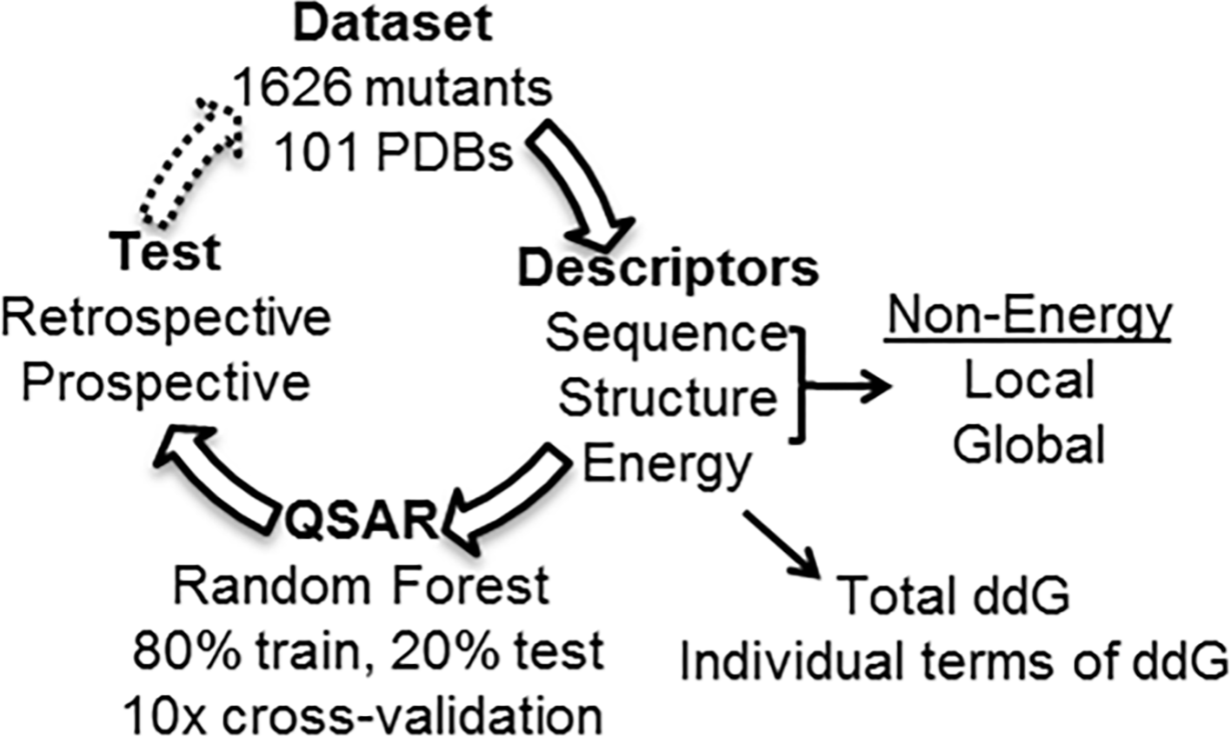
dTm model generation and prediction workflow.

The organization of this paper is as follows: energy and non-energy descriptors are derived from a high quality dataset of single-point mutants and used to train machine learning models that are then retrospectively and prospectively tested. The intent of this is to increase the available data used to generate dTm prediction models. (Fig 1) Retrospective and cross-validated results for each model were shown to be similar. Top performing models were trained with amino acid, structure, and free energy terms, with the best model (Cart_tiE_ALG) having a higher linear correlation (r^2^) (0.46) than existing machine learning models. (Table 1) Training a model with all descriptors (ALL) used in this study resulted in an r^2^ of 0.52 and a MAE of 2.67°C. (Table A in S1 Table) We find that training a machine learning model with a small list of amino acid and local structure descriptors (AL) provides similar results in 2–6 orders of magnitude less time than more complicated physics-based descriptors used to train existing machine learning models. (Table 1) This simple model was tested by performing a blind prospective study choosing 96 predicted mutants from all possible mutants of a protein not in the training dataset, the enzyme Guanylate Kinase, only taking into account the sequence location of each mutant. From this blind prospective study, we determine the shortcomings of the simple model and find incorporating additional terms that describe the environment of the mutant improved performance. However, we suspect improvement is limited by model applicability domain; which is assessed by comparing the variance of the global random forest model ensemble predictions and the variance of the cross-validation models. In our discussion we urge the community to systematically design protein mutants to increase and diversify the existing dTm datasets in the literature.

## Materials and methods

### Benchmark dataset

**Fig 2:**
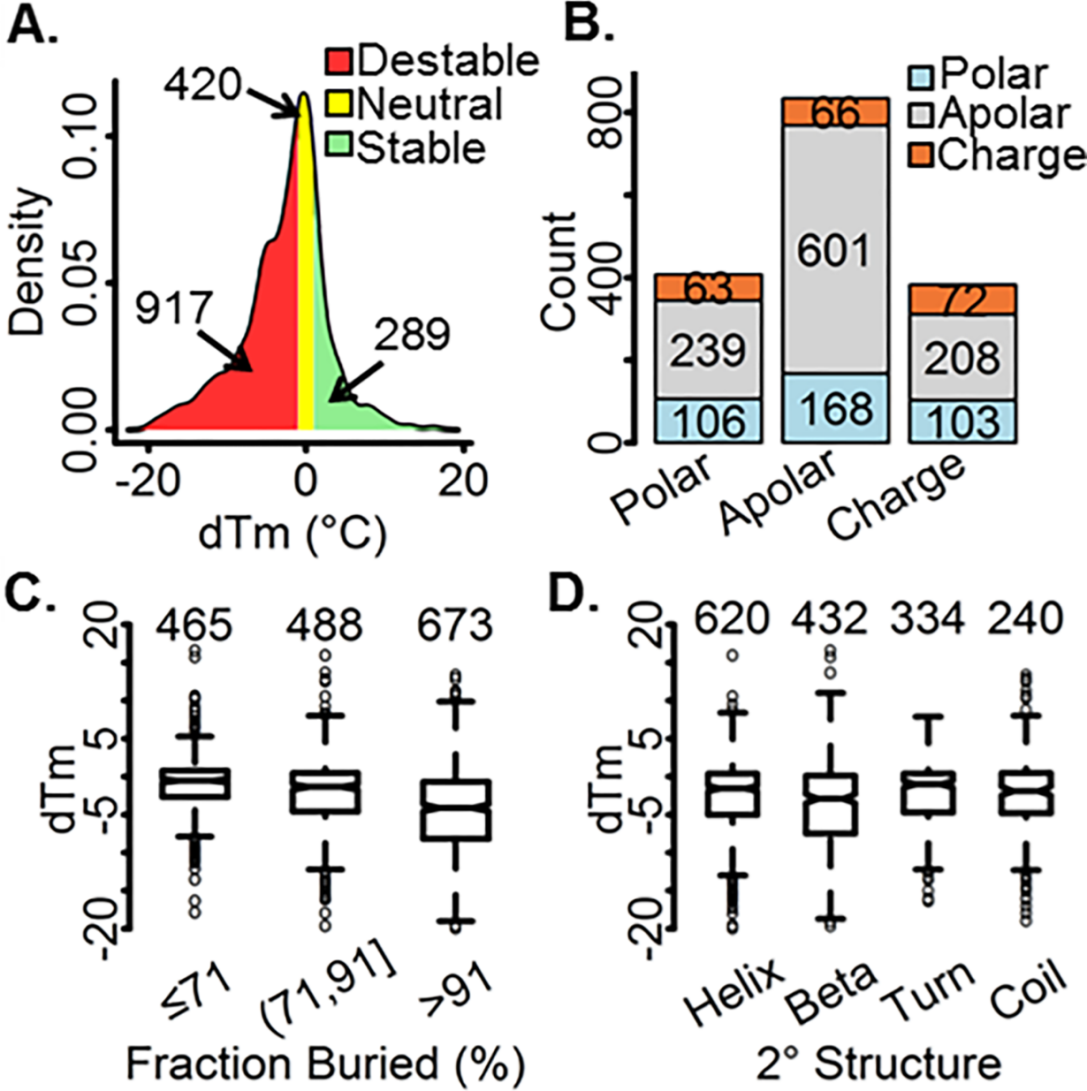
dTm dataset statistics. A) dTm density distribution and count of stabilizing (> 1°C), neutral ([–1,1]°C), and destabilizing (< -1°C) mutants, B) Wild-type to mutant counts, grouped by residue type (wt: x-axes, mut: color), C-D) Box plot of wild-type amino acid fraction buried and secondary structure, respectively.

The benchmark dataset used for model generation is that from Pucci et al. [35] and includes 1626 high-quality curated single-point mutants from soluble proteins that have experimental dTm values in physiologically relevant conditions and reported PDB structures. 289 of the mutants are stabilizing (dTm > 1°C), 917 are destabilizing (dTm< -1°C) and 420 are neutral (-1 ≤ dTm ≤ 1°C). (Fig 2A) Mutations are predominantly from apolar residues to apolar (601), polar (168) or charged (66) residues. (Fig 2B) dTm is confirmed [35] to significantly decrease as the fraction buried of the wild-type residue increases, where the median values were -0.5, -1.3, -4°C for mutants with ≤ 71, (71, 91), and > 91% fraction buried, respectively. (Fig 2C) This is to say, mutations at the surface of proteins have less effect. Residues most often are in a helix conformation (620). Change in protein thermostability is found to be more sensitive to mutations in beta conformation. (Fig 2D)

### Structure preparation for simulation calculation

Solvent and ligands were stripped from all structures 101 x-ray structures prior to mutagenesis. All x-ray structures used for the prediction of ddG of unfolding were prepared using the default parameters of each software package.

#### MOE

MOE 2016.0802 [36] was used. Structures were prepared using the standard Protonate 3D options, any issues were corrected with built-in structure preparation, and the system was initially parameterized with the standard Amber10 EHT force field. Site-directed mutagenesis was performed using Residue Scan with the default settings. Conformational search, using the amber10 rotamer library, was enabled using a cutoff distance of 4.5 Å for repacking, and mutation refinement was selected. Energy was calculated with and without implicit solvent enabled using the Generalized Born model (GB/VI) and results without implicit solvent are shown.

#### Rosetta

Rosetta ddG_monomer [37] and cartesian_ddg [38] applications were used. Structures were minimized prior to running each application with the default settings. ddG_monomer (Mono) was run with high resolution protocol flags enabled and cartesian_ddg (Cart) application was run with three iterations with the following standard flags specified: -fa_max_dis 9.0, -beta_cart weights, and -beta_nov16_cart as the scoring function.

#### Bioluminate

The residue scanning task of Schrödinger’s software Bioluminate (BL) version 2017–1 [39] was implemented using Prime Residue refinement, a standard cut-off distance of 0 Å, the OPLS2005 force-field, and the solvent model vsgb2.0. Energy descriptors were retrieved using the proplister command and the r_bioluminate terms for total (stability (ddG)) and individual (solv_gb, selfcont, lipo, vdw, ref, hbond, coulomb, packing, covalent, solv_sa) terms were used to train corresponding models.

#### Discovery studio

Structures were prepared with automatic structure preparation using the default CHARMm forcefield parameters prior to running the Calculate Mutation Energy protocols in pH-independent mode using the default CHARMm Polar H forcefield parameters within Discovery Studio 2017 R2 (DS). [40]

### Descriptors for machine learning

Amino Acid (A), local (L) and global (G) structure, and energy properties were used to generate machine learning models. Sequence descriptors were calculated for the wild-type (first), mutant (second), and change (diff) in amino acid properties alpha, beta, and coil propensity, aromaticity, branchness, bulkiness, charge, dG of transfer from octanol to water (Dg_O_W), flexibility, hydrophobicity, logD, molecular weight, mutability, pI, polarity, polarity2, Vdw Vol, and consensus hydrophobicity. [41] Local structure (L) descriptors were calculated for the wild-type residue. They include the secondary structure motif [41] and several forms of estimating solvent accessibility: total buried area, hydrophobic area, hydrophobic ratio, and fraction buried [42], where total buried area and fraction buried are defined as,

TotalArea=Solventaccessiblesurfaceareaofaresidueincludingthebackbone

Fractionburied=Solventaccessiblesurfaceareaofaresidueoccludedbyprotein/TotalArea

Global structure (G) descriptors were calculated using the proDesign.svl module from MOE 2016.0802: the change in the frictional and diffusion coefficient, radius of gyration, hydrodynamic radius, sedimentation constant, eccentricity, accessible surface area, hydrophobic surface area, hydrophilic surface area, volume, mobility, helix ratio, henry's function, net charge, dipole moment, hydrophobicity, zeta dipole and quadrupole moment, and charge and zeta potential at Debye length [36] between wild-type and mutant protein.

Energy descriptors were described previously and taken to be the total (totE), individual (indE), and the combination of total and individual (tiE) ddG energy terms for each molecular mechanics software, where each were defined as,

ddG(totE)=∑indE

tiE=totE+∑indE

A complete list of descriptors details can be found in the supplementary information.

### Random forest classifier

#### Model generation

Global QSPR models were generated using the R implementation of the random forest [43] method. Random forest is an ensemble learning method that generates predictions based on the mean prediction of many decision trees. It has many useful properties such as: giving very good predictions, insensitivity to correlated or useless descriptors, and few parameters to tune. It has previously been shown to be useful in predicting dTm values. [30] Models were built using a previous algorithm [44] with the default parameters of generating 100 trees without limiting the number of levels, choosing 1/3 of the descriptors at each branch point, and needing at least five molecules in a node for a branch point to be created. The Gini index was used as the splitting criterion.

Random forest provides, natively, an importance score for each descriptor used to train each model. This works by monitoring how the accuracy of the out-of-bag prediction works when descriptors are randomly reassigned to the wrong mutant. Individual descriptors may have non-zero importance purely by chance. To determine which descriptors were statistically significant, we noted the maximum descriptor importance that occurs when there is no real QSPR, i.e. in models where the dTm was randomly reassigned to the wrong mutant. Only descriptors that had an importance greater than this value were deemed useful.

#### Model validation

Prediction performances of the classifier were validated via 10 rounds of five-fold cross-validation using a 80/20 split. For each round of cross-validation, models were trained, without parameter optimization, on 80% of the benchmark training dataset and tested on the remaining 20% of the dataset. Other splits were also tried, resulting in similar statistics (results not shown). Statistics were derived from the results of the 10 cross-validated models. Methods for externally validating the global models using independent datasets derived from the literature and Guanylate Kinase are found in the Retrospective model validation and Blind prospective model validation sections, respectively.

### Performance metrics

#### Regression

Model performance was measured using the Pearson linear correlation coefficient (r^2^), the Spearman rank correlation coefficient (ρ^2^) and differences between observed dTm and predicted dTm were measured using the mean absolute error (MAE),

$$MAE=\frac{\sum_{i}^{N}\left|{predicted}_{i}-{observed}_{i}\right|}{N}$$

where i is the mutant dTm and N is the total number of mutants.

#### Classification

Categorical analysis was performed based on a 2X2 confusion matrix where stabilizing mutants were classified as dTm > 1 and destabilizing mutants as dtm < = 1 for simplicity. For ddG stability classification, stable mutants were classified as stabilizing if ddG < -1 and destabilizing if ddG > = -1. Accuracy (1), true positive rate (2), and false positive rate (3) were reported to describe model classification performance.

Accuracy(Q)=(TP+TN)/(TP+TN+FP+FN) (1)

TruePositiveRate(TPR)=TP/(TP+TN) (2)

FalsePositiveRate(FPR)=FP/(FP+TN) (3)

where TP is the number of true-positives, FP is the number of false positives.

Overall model performance for categorizing stability was taken to be the Area Under the Receiver Operating Characteristic (ROC) Curve (AUC), where

AUC>=0.9→excellentprediction

AUC>=0.8→goodprediction

AUC<=0.7→mediocreprediction

AUC<=0.5→randomprediction

#### Bootstrap analysis

Bootstrap analysis was done to determine whether the mean goodness of fit metrics (r^2^, MAE, etc.) among the models were truly different. For each bootstrapping trial, goodness of fit metrics over the cross-validation predictions were sampled with replacement 10 times, and averaged. There were 1000 bootstrap trials. Model A was considered significantly better than model B if the average goodness of fit metric for A was better than for B in >95% of the bootstrap trials.

### Applicability domain

The applicability domain of a QSPR model is the use case or query molecule space a model can reliably predict. A diverse set of approaches have been developed to determine if a query molecule is in the AD of a model. [45–48] Generally, AD approaches can be grouped into two categories: 1) assessing reliability based on a defined chemical descriptor space overlap between the training set and query molecule (i.e. outlier detection) [47] and 2) estimating model prediction uncertainty of a query molecule [48,49]. Outlier detection, when comparing multi-variate distributions, becomes problematic when nonlinear relationships exists between descriptors and when assessing prediction reliability at distribution boundaries. Rather, we use an estimate of uncertainty approach, specific to random forest, which does not need a predefined descriptor space overlap and takes into account nonlinear relationships between variables. [50] Briefly, the prediction is more reliable and considered in the AD of a model if the variance of the predicted property among the trees of the global model is less than the variance of the cross-validated models.

### Prospective model validation

#### Mutant selection and structure generation

We chose Guanylate Kinase (GK) from *Branchiostoma Floridae* (UNIPROT: C3YEM4_BRAFL) as a model system for prospective prediction because it is monomeric, highly expressed, easily purified, and displays two state-folding. More importantly, GK is not in the original set and has only ~20% sequence identity and ~70% homology to the closest protein in the dataset (Adenylate Kinase, pdbId 1AKY). The template sequence used in this research also incorporated seven mutations R11T, S24T, L54E, Y59F, I86L, S99A, and K138E which were identified during an evolution campaign to alter guanylate kinase function. Having a sequence identity of ~56% to mouse GK, the pdbId 1LVG was used as the structure template to generate the homology model using MOE with standard simulation parameters. The crystallographic ligands ADP and GMP were also modeled to generate a similar induced fit.

#### Construction of guanylate kinase genes

The gene for GK-RD3 with a c-terminal hexa-histidine tag was synthesized (GenScript, Piscataway, NJ, USA) and cloned into expression vector pCK110900 [51] under the control of a lac promoter. The genes for each of the GK mutants were produced by site directed mutagenesis (GenScript, Piscataway, NJ, USA) using GK-RD3 as the template dna. All constructs were transformed into E. coli W3110 and stored as glycerol stocks in 96 well plate format.

#### Guanylate kinase expression and purification

The guanylate kinase clones were expressed and purified from E. coli W3110 in 96 well plate format. To generate overnight saturated cultures, 10 ul of the GK clones as glycerol stock were inoculated into shallow 96 well plates with 190 ul of Lysogeny Broth (34 μg/mL Chloramphenicol and 1% Glucose). The cultures were shaken overnight (200 rpm, 30°C, and 85% humidity). The plates were inspected to ensure even growth at each well and confirmed by measuring absorbance of a 1:10 dilution at A600. To generate expression cultures, 10 ul of each overnight cell culture was transferred to a 96 deep well plate with 390 ul of Terrific Broth (34 ug/ml chloramphenicol). The plates were incubated in a Kuhner shaker for 3 hours (250 rpm, 30°C, 85% humidity) until OD600 reached ~0.8. To induce the expression culture, 10 mM Isopropyl thiogalactoside (IPTG) was introduced to the deep well plates and they were returned to the Kuhner shaker to continue expression overnight. The next morning, cells were harvested in the deep well plates by centrifugation (4°C, 15 min, 4000 rpm) and cell pellets were stored at -80°C for further use (at least for 2 hours).

To lyse the harvested cells, cell pellets were re-suspended in 50mm sodium phosphate pH 7.4, 300 mM NaCl, 1 mg/ml of lysozyme and 0.5 mg/ml of PMBS. Cell lysis was allowed to proceed for 2 hours at 25°C with shaking at 1000 rpm on a table top orbital shaker. After lysis, insoluble cell debris was removed by centrifugation (4°C, 15 min, 4000 rpm). A total of 200 μL of supernatant of each mutant was combined with equilibration buffer (50 mM sodium phosphate pH 7.4, 300 mM NaCl) in a 1:1 ratio. The supernatants were loaded onto HisPur Ni-NTA 96-well spin plates (Thermo Scientific Cat# 88230) in 100 μL increments followed by centrifugation at 1000 X g for 1 min (this was repeated four times). The HisPur plates were then washed three times by applying 250μL wash buffer (50mm sodium phosphate, 300 mM NaCl pH 7.5, 50 mM Imidazole) and centrifuging for 1 min. The GK proteins were finally eluted from the plate by applying 200 μL elution buffer (50 mM sodium phosphate, 300 mM NaCl pH 7.5, 250 mM Imidazole) and centrifuging for 1 min at 1000 X g. All 96 well plate operations were performed using Biomek FX Instruments.

#### Guanylate Kinase (GK) concentration and purity measurements

Concentration and purity from the whole cell soluble lysates and purified proteins through the spin plate in all GK mutants were measured, analyzed and validated by LabChip GXII Touch (PerkinElmer, Inc).

#### Thermostability measurements

To measure protein stability, 12.5 μL of each purified GK mutant was mixed with 5 μL of protein thermal shift buffer (1 M potassium phosphate pH 7.0) and 2.5 ul of 8X protein thermal shift membrane dye (M46045). Protein stability was measured in 96 well format using a Quant Studio 3 qPCR instrument to ramp sample temperatures from 4 to 95°C at 0.15°C/sec while monitoring the absorbance of the protein thermal shift membrane dye. The Protein Thermal Shift (ThermoFisher Scientific) software was used to analyze data and calculate Tm values. All the products above were purchased from Thermal Fisher Scientific.

## Results

### Expanded study of calculated ddG vs. dTm

Predicting mutant dTm classification using ddG values calculated from commercial molecular mechanics packages is an attractive “point-and-click” method for qualitatively triaging mutations without the need for the user to calculate additional descriptors. Ford et al. [23], using a set of 62 mutations, investigated the correlation of the MM packages (Bioluminate (BL), Discovery Studio (DS), Schrodinger FEP+, and MOE) calculated ddG values to experimentally determined dTm and found a range of linear (r^2^, 0.25–0.50) and rank order (ρ^2^, - 0.17–0.53) correlations, where FEP+ was clearly more favorable. (Table 2) Using FEP+ to calculate ddG values for the entire dataset set used is in this paper (1626 mutants) is impractical due mainly to high computational costs and difficulties with predicting ddG of charge changing mutations. [52] Therefore, we decided to test the MM packages previously used (BL, DS, and MOE) against a larger dataset and expand the set of MM packages to include two Rosetta ddG calculation methods: ddG_monomer (Mono) and Cartesian_ddG (Cart). We used each software package in the “out-of-the-box” configuration. Generally, testing on a larger dataset compared to Ford et al. [23] led to a large decrease in linear correlation (e.g. 0.28 -> 0.03 for BL), a large increase in accuracy (e.g 0.25 -> 0.82 for MOE), and similar rank correlations (Table 2). Metric differences may be attributable to the ddG energy cut-off used to classify stability, where Ford et al. [23] derived bounds between destable, neutral, and stable ddG values, for each MM package separately, based on the experimental dTm range observed and we used a conservative cut-off of 1 kcal/mol for each MM package. Additionally, Ford et al. [23] carefully chose a dataset with a balanced distribution of stabilizing and destabilizing mutants, whereas our dataset was predominantly biased towards destabilizing mutants. Regardless of the differences in ddG classification and dataset distributions used in each study, the overall performance of each MM package was poor, with Cartesian_ddG potentially being the most robust method able to capture a diverse dataset two orders of magnitude slower than most other methods. (Table 2) Although not within the scope of this paper, an in-depth analysis of the performance of each MM package for the different mutant types may help understand the shortcomings of each “out-of-the-box” software configuration. In this respect, an important note to mention is that the ddG terms for MOE and DS are derived from linear regression models that have been trained on experimental ddG values.

**Table 2:** Statistics of calculated ddG versus experimental dTm values where r^2^, ρ^2^, and Q is linear and rank correlation and accuracy, respectively.

Model	Min./Mut.	^(a)^r^2^	^(b)^r^2^	^(a)^ρ^2^	^(b)^ρ^2^	^(a)^Q	^(b)^Q
BL	0.50	0.03	0.28	0.23	0.19	0.74	0.50
Cart	20	0.27	NA	0.31	NA	0.78	NA
DS	0.80	0.09	0.33	0.22	0.21	0.81	0.33
MOE	0.53	0.16	0.25	0.14	0.17	0.82	0.25
Mono	101	0.08	NA	0.20	NA	0.78	NA

^(a)^ This paper tested 1626 mutants

^(b)^ Ford et al. tested 62 mutants [23]

### Training with ddG functional terms

In addition to the final ddG value, molecular mechanics packages also output the ddG functional terms that make-up the final ddG calculation. Rather than using one term (ddG) known to correlate with dTm to predict dTm, we expected that training models with other energy terms known to contribute to dTm (e.g. hydrogen bonding) would lead to an increase in model predictivity. In essence, we utilized the terms of the ddG functional to retrain the ddG functional to predict experimental dTm values. We investigated three models: training with 1) only the final ddG calculation value (totE), 2) only the individual functional terms that make up the final ddG term (indE), and 3) both the final ddG term and the individual terms of the ddG functional (tiE = totE + indE). Models trained on tiE terms generally performed significantly better (p-value < 0.05) than both totE and indE models (Table A in S1 Table, Table 3).

**Table 3:** Statistics of cross validated models trained with tiE energy terms versus experimental dTm values, where r^2^, ρ^2^, and Q is linear and rank correlation and accuracy, respectively.

Model	Min./Mut.	r^2^	ρ^2^	MAE (°C)	MCC
BL	1.50	0.32	0.26	3.18	0.23
Cart	21	0.39	0.32	3.00	0.31
DS	1.80	0.21	0.19	3.49	0.20
MOE	1.53	0.40	0.34	2.96	0.34
Mono	102	0.33	0.26	3.19	0.31

Comparing the cross-validated results of the tiE models, the general trend was MOE > Cart > Mono > BL > DS (Table 3) with MOE having the highest linear (0.40), and rank (0.34) correlation, lowest MAE (2.96°C), and highest Matthews Correlation Coefficient (MCC) (0.34). Noteworthy, due to mutant imbalance (1337 destabilizing vs 289 stabilizing), Matthews Correlation Coefficient is a more applicable measure of dTm classification quality than accuracy for this study because it is generally regarded as insensitive to dataset imbalances. Calculation speed for each tiE model was compared by looking at the average time per mutant which ranged from 0.50–101 minutes/mutant and trends from quickest to slowest were BL > MOE > DS > Cart > Mono. For reference, training machine learning models only with ddG values (totE) led to a significant rank order (ρ^2^) improvement, as per a two-tailed Wilcoxon rank sum test with a p-value < 0.05, over MM models comparing calculated ddG values directly to dTm (Table 2), where MOE (0.44) was the top performer (Table A in S1 Table). Importantly, training and validating a machine learning model added a minimal amount of time (~1 minute) compared to only executing the MM packages. (Table A in S1 Table, Table 3)

### Simple physicochemical descriptors

**Fig 3:**
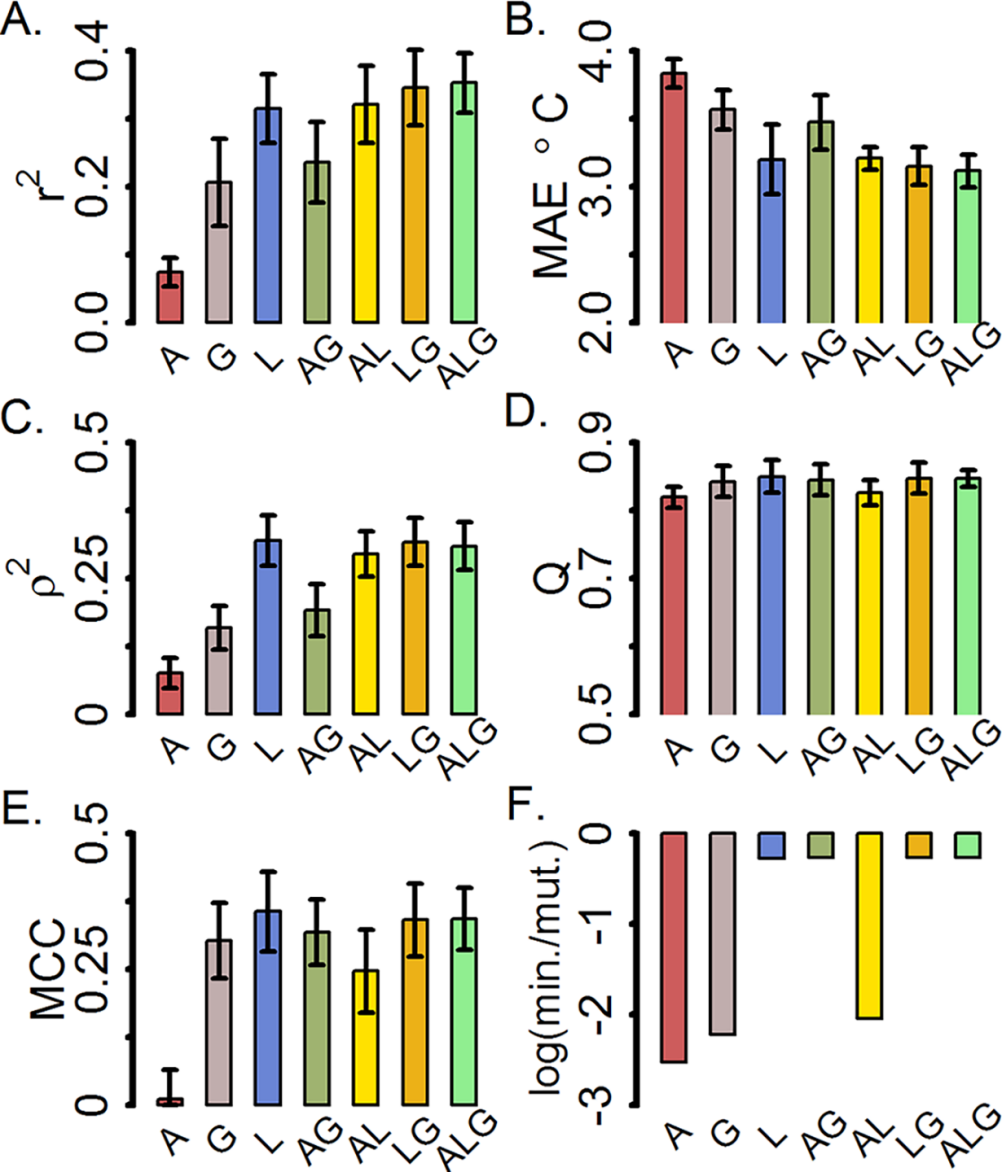
Goodness of fit metrics of cross-validated models trained on only amino acid (A), local (L) or global (G) physical descriptors, or a combination of amino acid and physical descriptor types (e.g. model ALG is trained on all three descriptor types). A) Pearson linear correlation, B) Mean absolute error, C) Spearman rank correlation, D-E) Accuracy and Matthews correlation coefficient computed from each confusion matrix, and F) Log base 10 of the calculation time per mutant. Bar height and error bars represent the mean and standard deviation of the 10 cross-validated tests for each model.

Previous dTm prediction models have indirectly examined the effectiveness of using mutant descriptors as descriptors for building models. Statistical potentials have been derived using a limited number of properties around the mutant such as solvent accessibility, secondary structure, hydrogen bonding patterns, and other local interactions. [27,29,31] However, whether training a model using solvent accessibility by itself is able to enhance dTm predictive capabilities has not been performed. Jia et al. [30] trained models using amino acid properties, secondary structure, solvent accessibility, and calculated ddG. However, they did not explicitly show the performance of models without energy terms. We directly examined the predictive performance of models trained on combinations of three types of non-energy mutant descriptors easily calculable from a protein structure: 1) amino acid (A), 2) local structural (L), and 3) global structural (G) properties. Our aim was to understand the limitations of simple models before employing more complex solutions to predict dTm. Testing the hypothesis that dTm is context dependent, we decided to separately test descriptor sets that do or do not include information about the environment of the mutant. As a baseline descriptor type, we choose to include amino acid properties, precalculated from the literature, that describe the chemical identity of the mutant wild-type, mutant, and substitution difference. [41] Amino acid properties do not uniquely describe the environment of a mutant, i.e. a valine to an alanine substitution will have the same value regardless of position of the mutated residue in structure. As expected a model trained with A descriptors alone had the lowest regression correlation (Fig 3A), highest mean absolute error (Fig 3B), the lowest rank correlation (Fig 3C), the lowest accuracy (Fig 3D) and lowest Matthews correlation coefficient (Fig 3E) mean cross-validated values compared to models trained with descriptors that specifically incorporate environment information (L, G, AG, AL, LG, ALG). Bootstrap analysis on the 10 cross-validation means for each goodness of fit metric confirmed models trained on A descriptors performed, with confidence, worse than the structure models with at least 95% probability. Importantly, the model trained with A descriptors also serves as a calculation baseline having a minimum time of 3*10^−3^ minutes/mutant required to calculate descriptors. (Fig 3F)

### Local descriptors

Incorporating descriptors specific to the environment of the mutant residue was expected to enhance prediction of dTm. Models trained on local structural (L) descriptors were observed to perform significantly better (>97% confidence by bootstrapping) than models trained on global structural (G) and amino acid (A) descriptors when comparing all goodness of fit metrics (Fig 3A–3E). Combining A, L, and G (ALG) descriptors significantly (> 95%) improved model performance over models AG and G and performed similarly compared to models L, AL, and LG for most goodness of fit metrics. Interesting to note, the MCC for ALG (0.34) was significantly higher than AL (0.25). In general, models trained with local structure descriptors lead to top performance and are up to two orders of magnitude faster (L 0.003 min/mut) than models including global descriptors (Fig 3E).

### Energy and non-energy descriptors

**Fig 4:**
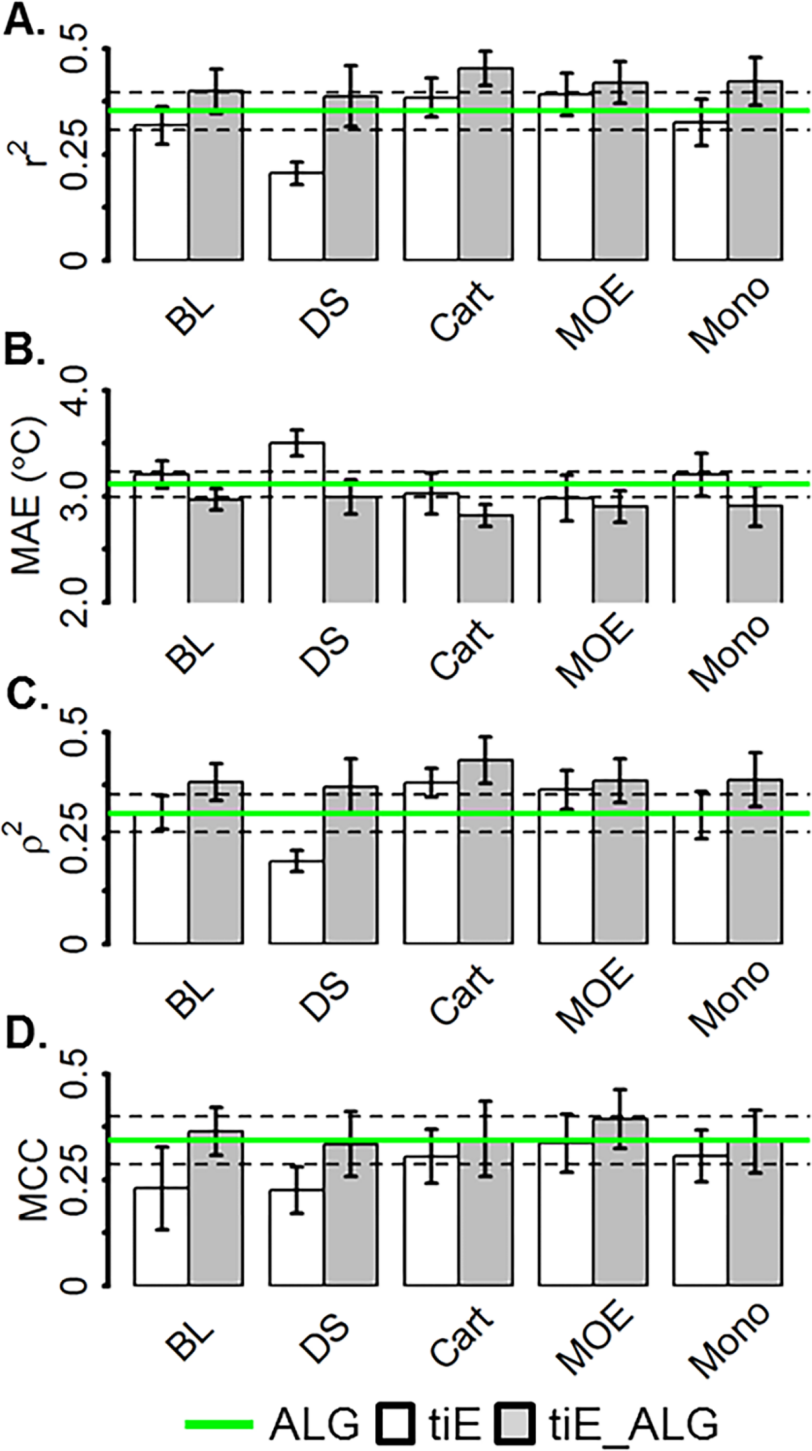
Goodness of fit metrics of cross-validated models trained on amino acid and local and global physical (ALG), or calculated total energy and individual energy of unfolding (tiE) or both (tiE_ALG) protein descriptors. A) Pearson’s linear correlation, B) Mean absolute error, C) Spearman rank correlation, D) Matthews correlation coefficient computed from each confusion matrix. Dashed lines are ALG standard deviations from each metric mean. The colors white, grey, and green represent the mean metric for the tiE, tiE_ALG, and ALG models. Bar height and error bars/dotted lines represent the mean and standard deviation of the 10 cross-validated tests for each model, respectively.

Balance between cost and model predictivity is an important deciding factor when considering which descriptors to use when building a building a model of dTm. Therefore, we ask if including costly molecular mechanics energy descriptors (tiE) significantly enhances performance compared to models with only less costly physical descriptors (ALG). Model ALG, which performed as well as models L and AL, was compared to the results of models trained with only tiE descriptors (e.g. BL_tiE_) and both tiE and ALG (i.e. BL_tiE_ALG_) descriptors (Fig 4A–4D).

Bootstrapping analysis showed that ALG performed significantly (> 95%) better than BL_tiE_ at improving the overall accuracy as measured by MCC (0.34, 0.23) and similarly for other statistics. Combining ALG and BL_tiE_ (BL_tiE_ALG_), significantly (> 99%) improved each metric over BL_tiE_ and significantly (> 98%) enhanced linear (0.40, 0.35) and rank (0.38, 0.31) correlation compared to ALG alone. ALG performed significantly (> 98%) better than DS_tiE_ for linear correlation (0.35, 0.21), mean absolute error (3.11, 3.51°C), rank correlation (0.30, 0.20), and overall accuracy as measured by MCC (0.34, 0.23). Combining DS_tiE_ and ALG (DS_tiE___ALG_) led to a significant (> 99%) rank correlation improvement over ALG alone (0.37, 0.31). Cart_tiE_ had a significantly (> 95%) higher linear (0.38, 0.35) and rank (0.38, 0.31) correlation than ALG. Training with Cart_tiE_ and ALG (Cart_tiE_ALG_) led to significant (> 99%) increases in linear (0.45, 0.35) and rank (0.43, 0.31) correlation and MAE (2.82, 3.12°C). MOE_tiE_ significantly (> 95%) outperformed ALG alone when comparing linear (0.39, 0.35) and rank (0.36, 0.31) correlation and MAE (2.98, 3.11°C). Combing ALG and MOE_tiE_ (MOE_tiE_ALG_) led to a significant increase (> 97%) for all statistics over ALG. Mono_tiE_ and ALG were observed to not be significantly different. Combining ALG and Mono_tiE_ (Mono_tiE_ALG_) led to a significant (> 99%) enhancement in linear (0.42, 0.35), and rank (0.39, 0.31) correlation and MAE (2.91, 3.12°C) compared to ALG alone.

In general, combining ALG and tiE energy descriptors led to a significant improvement over both the individual sets of descriptors. The time requirements for calculating each set of tiE energy descriptors versus the overall marginal improvement compared to ALG models maybe the deciding use factor for including tiE descriptors.

### Retrospective model validation

**Fig 5:**
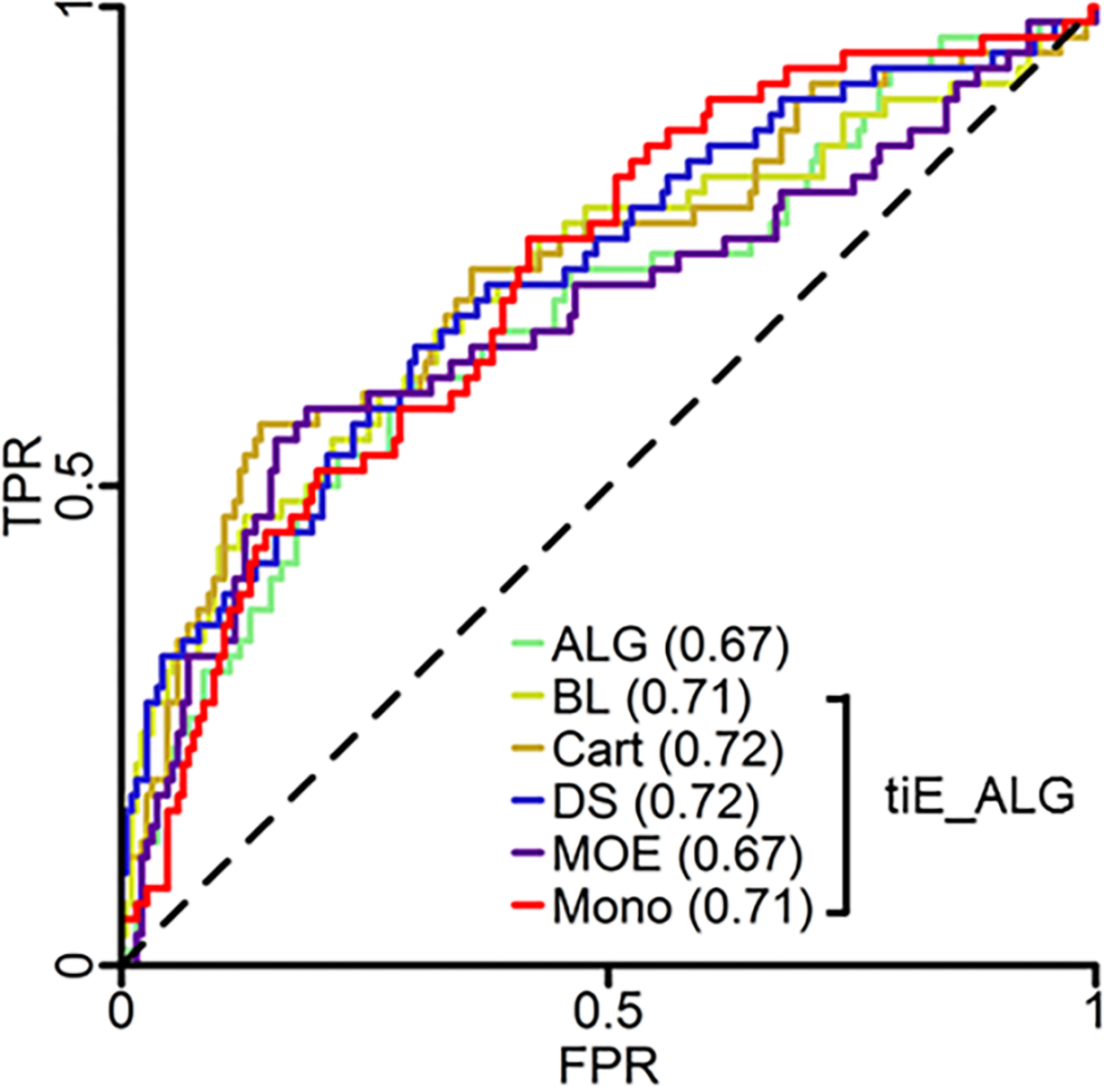
Retrospective study of global model performance. ROC plots of models trained on amino acid and local and global physical (ALG), and both ALG and total energy and individual energy of unfolding (tiE), for each MM software package, (tiE_ALG) protein descriptors. AUC values are in parenthesis. The black dotted line is the performance of a random model with an AUC of 0.50.

To further test the top performing global non-energy (ALG) and energy plus non-energy (tiE_ALG) models, a non-overlapping subset of 251 mutants between Jia et al. [30] and Pucci et al. [35] was retrospectively predicted (i.e. tested on existing data not in the benchmark training sets). This set contains 62 stabilizing and 189 destabilizing mutants (see supplementary information files for mutant information). Typically, when designing thermostable proteins, the predicted mutant stability class (stabilizing versus destabilizing) rather than the absolute dTm value is more important. Therefore, performance of each model was measured by ROC curves to gauge the ability to predict correctly mutant stability class. (Fig 5) Each model performed better than random and had a similar ROC curve behavior. Area under the ROC curve (AUC) values were mediocre and also similar, where the trend was Cart_tiE_ALG_ = DS_tiE_ALG_ > BL_tiE_ALG_ = Mono_tiE_ALG_ > ALG = MOE_tiE_ALG_. Compared to the cross-validated results, the prospective AUC results were similar although decreased for each model: ALG (0.72, 0.67), BL_tiE_ALG_ (0.76, 0.71), Cart_tiE_ALG_ (0.79, 0.72), DS_tiE_ALG_ (0.74, 0.72), MOE_tiE_ALG_ (0.75, 0.67), and Mono_tiE_ALG_ (0.77, 0.71) (Table A in S1 Table).

### Blind prospective model validation

**Fig 6:**
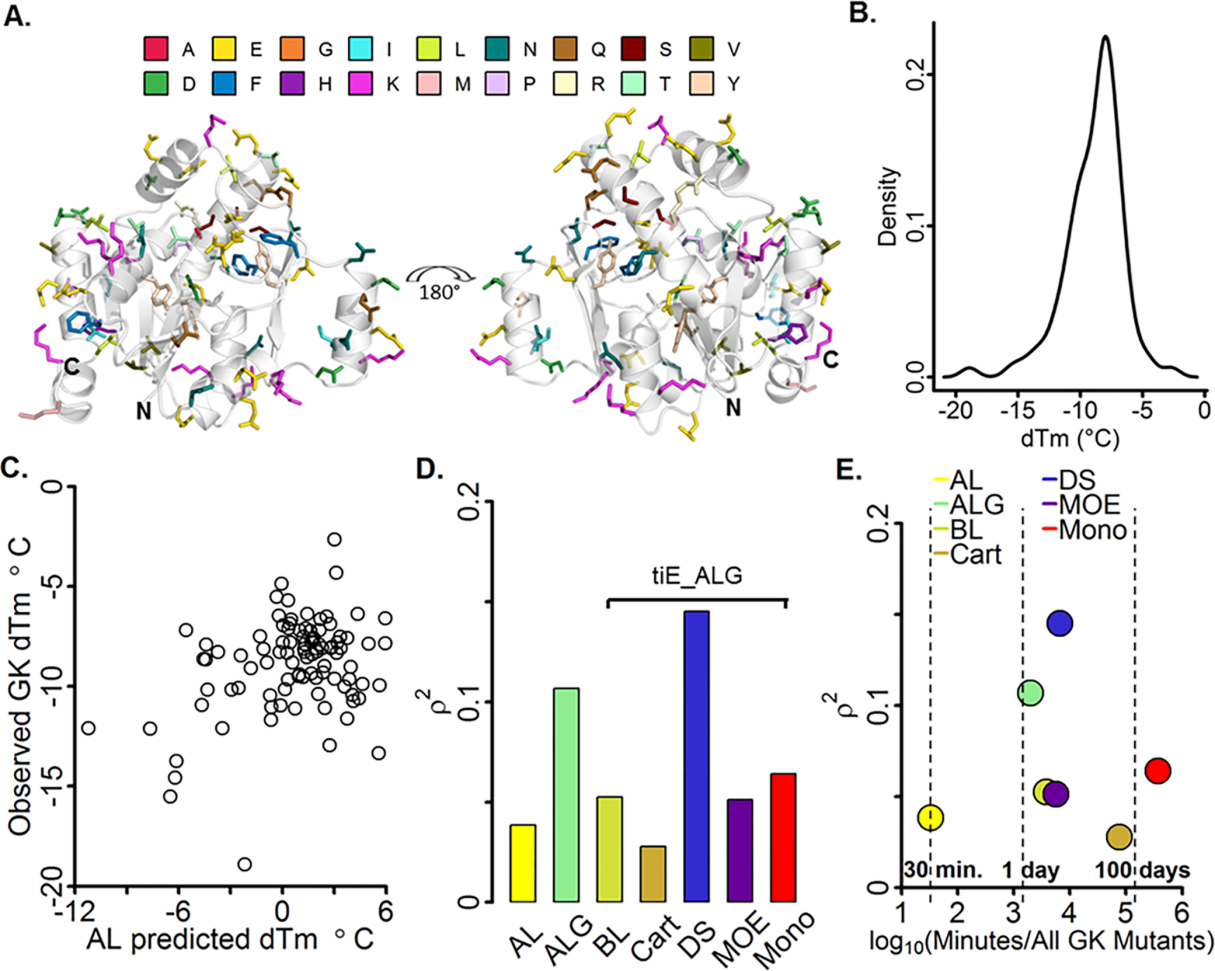
Blind prospective study of global models on GK mutants. A) Structure of Gkase with 96 mutants blindly selected to be stabilizing by the AL model represented as sticks and colored by residue type, B) Experimental dTm density distribution, C) Point plot of predicted versus observed dTm. D) Spearman rank correlation for each global model, with bar height calculated from the final predictions of each model, and E) Spearman rank correlation vs real elapsed time needed to compute the descriptor set for each non-energy (ALG) and non-energy plus energy (tiE_ALG) models for all possible single-point mutants of GK.

To our knowledge the external models shown in Table 1 have not been used or cited in literature to prospectively triage all single amino acid substitutions for the design of thermostable protein mutants. We choose 96 mutants (out of 191*19 possible) of Guanylate Kinase (GK) solely on the prediction of the AL model, which had a balance between calculation speed and cross-validation performance. Mutants were selected without reference to structural or sequence evolution conservation information to strictly measure model performance in a blind test. Although blind to structure and evolution, mutants were chosen to sample positions along the amino acid sequence of GK. Mutants predicted to be stabilizing (63) and destabilizing (33) were chosen and predominantly were located on the surface; where 56, 27, and 13 mutants had a fraction buried of ≤ 71, (71, 91], and > 91%, respectively. Of note, there were 21 charged to apolar mutants with fraction buried less than or equal to 71%. (Fig 6A)

Out of the 96 GK mutants selected by model AL none were experimentally observed to be stabilizing (Fig 6B and 6C). Even though no true-positive results were observed, selecting a model with a high rank order and quick calculation time is also important when minimizing the experimental cost of mutant triage. Comparing model AL predictions with GK experimental results revealed a 0.20 rank correlation, which is acceptable performance considering model AL cross-validation results (0.31). (Fig 6D) Calculating all AL descriptors for GK took ~29 minutes. (Fig 6E) A growing consensus from large-scale single-point random mutagenesis studies on proteins ranging from 30–450 amino acids [53–56] suggests stabilizing mutants are uncommon, however are estimated to occur in ~2% of cases. [57] Therefore, it is surprising that none of the 96 GK mutants (out of 3629 possible, ~2.6%) were stabilizing. We suspected AL performance of worse than random was due to model bias. We hypothesized model AL bias was due to applicability domain issues involving inadequate descriptor selection and limited mutant type and protein family of the underlying dataset.

### Rescue model performance

To help understand the limits of the AL model, we hypothesized that descriptors used in the AL model did not adequately describe the environment of the GK mutants and including environment relevant descriptors would enhance performance. With the majority of the GK mutants on the surface of the protein and a large percentage of them being charge to apolar (21), charge swapping (11) (i.e. negative to positive or vice versa), or charge neutralizing (8) we expected descriptors describing the change in surface interactions would enhance predictive performance. Although fraction buried, hydrophobic ratio and hydrophobic and total area were determined to be statistically important local (L) descriptors for predicting dTm, additional factors known to impact thermostability, such as changes in electrostatic, non-polar and hydrogen bonding interactions, were not taken into account. In an effort to rescue AL performance and utilize more complex models to further triage potential mutants, we selected descriptors that explicitly incorporate these changes. We included two types of descriptor sets to AL: 1) global descriptors (G) describing the overall change in protein properties, and 2) energy based descriptors (tiE) describing the change in free energy components upon amino acid mutation.

Evaluation of model G performance revealed the overall change in dipole moment, radius of gyration, volume and eccentricity were statistically important descriptors for predicting dTm. Changes in the dipole moment have been designed to enhance thermostability [18,58] and have been used to develop a method of predicting the change in thermostability by minimizing the dipole modulus. [59] Upon addition of model G descriptors to AL (ALG), an increase in rank order correlation (0.20 to 0.33) similar to the cross-validated results of ALG (0.33) (Fig 6D) was observed. As a point of reference, it would have taken an additional 1.38 days (Fig 6E) more than model AL for all ALG descriptors to be calculated for all possible single-point mutants of GK. The increased predictive performance of model ALG points to a potential for further filtering of mutants by only slightly increasing the cost of calculation. Although an improvement was observed with the addition of G descriptors, changes in the dipole moment itself have been shown by Wunderlich et al. [60], using a carefully selected set of charge changing mutants on the surface of a cold-shock protein, to not be entirely correlated with dTm and therefore suggests additional descriptors are needed to be accounted for to further enhance GK predictive performance.

To further improve GK predictivity, we expected combining tiE descriptors with ALG descriptors, which directly account for solvent, ionic, hydrophobic, and hydrogen bonding interactions, would further increase rank correlation. Analyzing the descriptor importance for each tiE model revealed that ddG is the most important descriptor for all models, and Van deer waals energy terms for BL and DS and the attractive Leonard Jones term for Cart and Mono were also significantly important. Although all tiE_ALG models predominantly outperformed or similarly performed in comparison to AL with respect to rank correlation, it is surprising that tiE_ALG models generally performed worse than or similar to ALG models. Model ALG had a higher rank correlation (0.33) than all tiE_ALG models (BL, 0.23; Cart, 0.17; MOE, 0.23; Mono, 0.25) except DS (0.38). Putting the increased performance of ALG into perspective with respect to time, calculating ALG descriptors for all 3629 GK single-amino acid mutants was 2-257x times faster (1.4 days) than calculating all the descriptors for each tiE_ALG model: BL, 2.6; Cart, 52.9; DS, 4.5 MOE, 3.9; and Mono, 257.1 days. (Fig 6D and 6E) The predominant reduction in performance metrics when including tiE terms to ALG and the overall poor performance of each model suggests a more systemic issue is present, rather than an insufficient descriptor selection, in the case of predicting the GK dataset results. In the next section we suggest that the underlying mutant and dTm dataset distribution limits the applicability domain of each model.

### Applicability domain of models

**Fig 7:**
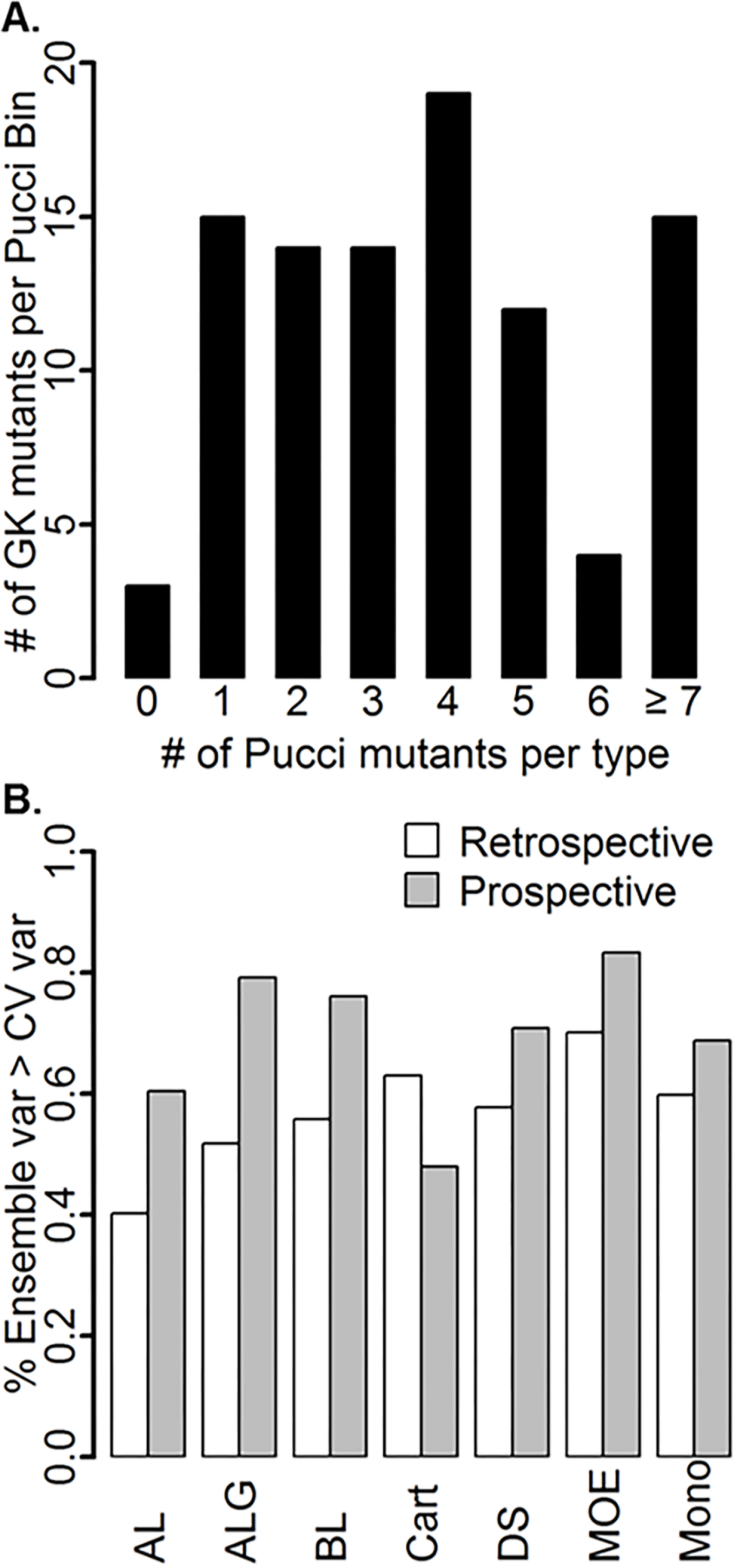
Applicability domain metrics of global models. A) Binned distributions of Pucci et al. mutant types versus number of GK mutants in each bin, and B) Percentage of mutants with higher ensemble variance than cross-validated variance for the retrospective (non-overlapping dataset of Jia et al. [30] and Pucci et al. [35]) and prospective (GK) models. Note that MM models are trained with tiE_ALG descriptors.

**Fig 8:**
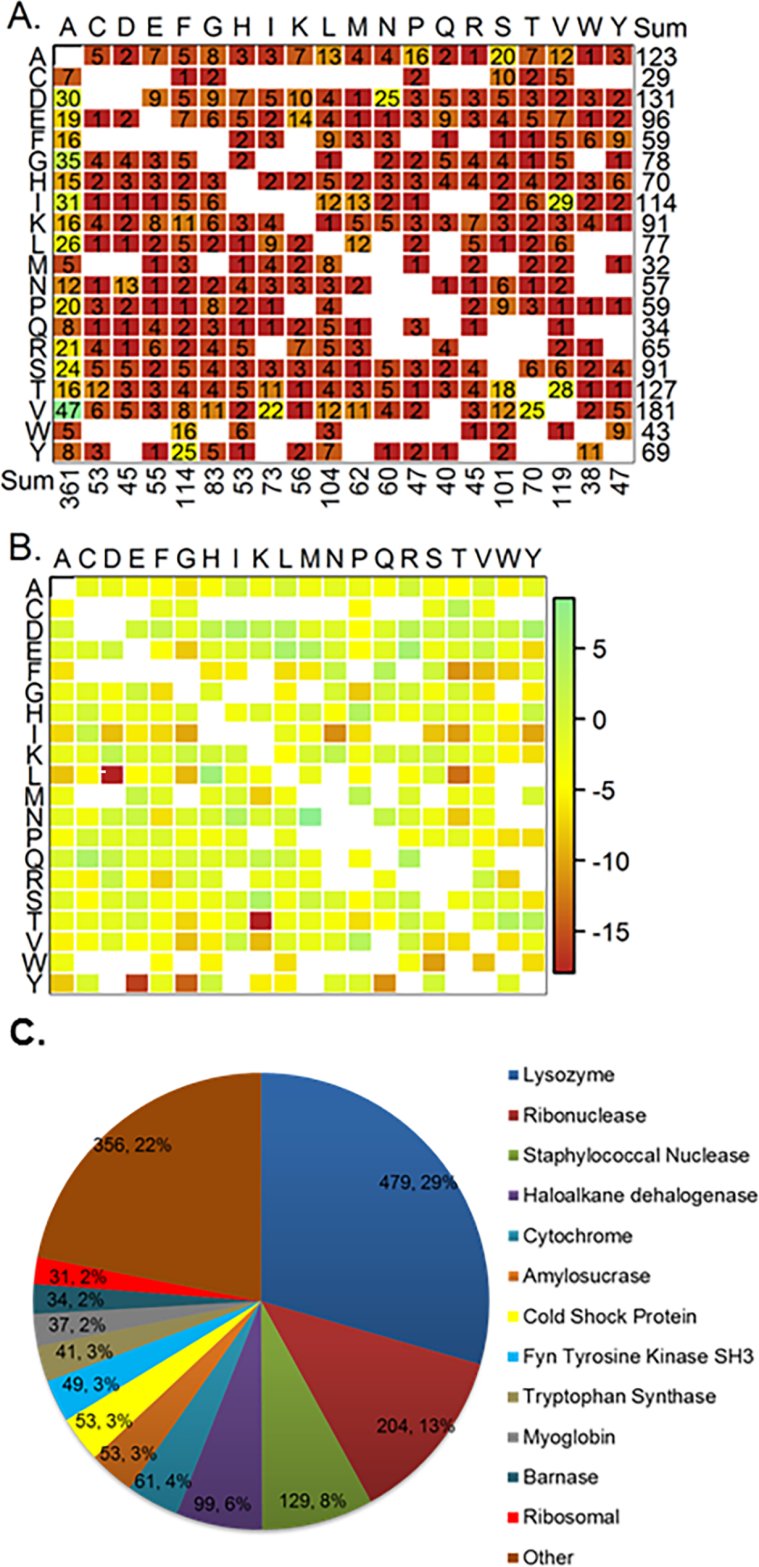
Distribution statistics of training dataset. A) Mutant counts per wild-type (wt) -> mutant (mut) amino acid substitutions and B) Mutant type average dTm per wt -> mut substitutions, and C) Mutations per protein family.

A closer look at the underlying dataset used to generate the energy and non-energy models within this paper makes it apparent that the majority of GK mutants may not be in the applicability domain of the models. Immediately, it is clear that each model has seen only a few examples of mutant types that are present in the GK query and therefore the models cannot be deemed reliable to predict the GK mutants selected, as proposed by the TARDIS method of assessing AD [61] The majority of GK mutant types (65) were observed in the Pucci et al. training dataset less than five times and several (3) were not observed entirely. (Fig 7A) For instance, mutant type E->L was observed four times in the dataset with an average dTm of 5°C (Fig 8A and 8B), whereas 11 GK mutants were chosen that had an average dTm of -9.3°C (See supplementary information files). Investigating applicability domain metrics further, we calculated the percentage of mutants with higher ensemble prediction variances than the variance of the cross-validated (CV) model, which has been shown to be an effective measure of applicability domain. [48,49] Mutants with higher predicted ensemble variance than the CV model variance were deemed likely to be outside the model prediction domain and not reliable. Values ranged from 48 to 83 percent of mutants had a higher ensemble variance than the CV model variance for GK predictions, where the order was Cart < AL < Mono < DS < BL < ALG < MOE (Fig 7B). For comparison, the retrospective study showed a general decrease in the percentage of mutants with higher ensemble variance than the CV model variance, except for Cart, suggesting the retrospective data is closer to the applicability domain of the training set than the prospective GK mutants (Fig 7B). A confounding issue with the Pucci et al. dataset is that the majority of the mutants are from the lysozyme (29.5%), ribonuclease (12.5%), staphylococcal nuclease (7.9%), and haloalkane dehalogenase (6.1%) families and the closest protein to GK (Adenylate Kinase) makes up only 0.6% (10 mutants) of the protein distribution. (Fig 8C) Additionally, out of the 19*19 possible mutations in the Pucci et al. dataset, only 80% of all mutant types were captured and the majority was observed less than five times and was predominantly from alanine scans. (Fig 8A)

## Discussion

Machine learning is a promising approach to building models that predict the effects of single-point mutations on protein thermostability. QSPR models in the literature and herein were shown to have similar performance despite being built using a diverse set of machine learning methods and a wide variety of descriptors, both simple and complex. Inclusion of even more complex descriptors, e.g. conformational flexibility [21,62–65], may enhance predictive capabilities, however the limited and unbalanced experimental dataset available to train models is shown, herein, to be a major obstacle for prospective predictions. Our results suggest that most published models built with the available datasets would suffer from the same ill fate of having narrow applicability domain (i.e. poor prediction performance on mutations not in or poorly represented in the training set and from proteins with low homology to ones in the training set). Deriving narrow training sets to probe the usefulness of each model to specifically predict mutants in a particular secondary structure, amount buried [28], or mutant type (i.e. wild-type -> ALA), is an appealing idea and may elicit a deeper understanding of the applicability of the underlying dataset, however, the insufficient nature of the data still proves to be a problem. QSPR modeling has been successfully applied to many fields, including quantum [66], materials [67], and drug discovery [68] chemistry, and typically have much larger training datasets on the order of 10^4^−10^5^ data points. One proposal for increasing the mutation training dataset size is to assume the mutant -> wild-type has the opposite dTm as the wild-type -> mutant. [30] More practically, experimental methods used to generate large-scale stability datasets for proteins [69,70] maybe amenable to design for generating large-scale dTm datasets to enhance and further validate the available QSPR methods. We suggest the community to critically and systematically design mutant datasets that fill out gaps in the proTherm [71] dataset to cover the distribution of each known protein family and potential mutants. Sadly, proTherm does not seem to be regularly updated, and new databases of thermostability data [72,73] are still too small to be useful. In real life project support, you would not use these trained models in a blind prospective test. Mutant triaging would be complimented by visual inspection and physicochemical intuition (i.e. not disrupting a salt-bridge). In addition to intuition, prior to mutant selection, the use of applicability domain metrics such as ensemble variance, or a metric akin to molecular similarity scores [74] could be generated to compare mutant microenvironments [75,76] to discriminate prediction reliability for each mutant.

## Conclusion

A diverse set of descriptors has been used to train QSPR models to predict dTm. Models trained on simple physicochemical descriptors performed as well as more complicated models in the literature. This paper finds that training models using ddG functional terms improves dTm predictions and including context relevant descriptors specific for the mutant environment improves predictive capabilities. Significantly, retrospective and prospective studies using existing and generated datasets highlight the insufficiencies in the diversity and size of the mutation datasets that currently exist. We urge the community to develop methods for generating larger dTm datasets.

## Supporting information

S1 File Datasets and descriptor information. (XLS)

S1 Table Table A. Statistics of dTm QSPR model predictions.
(DOC)
