# Supplementary material for: Role of simple descriptors and applicability domain in predicting change in protein thermostability
Source: PLoS One. 2018 Sep 7;13(9):e0203819. doi: 10.1371/journal.pone.0203819 (PMC6128648; doi:10.1371/journal.pone.0203819)
Supplement: S1 Table — Table A. Statistics of dTm QSPR model predictions. (DOC) [file pone.0203819.s002.doc]

**Supporting Information**

**Role of simple descriptors and applicability domain in predicting change in protein thermostability**

**Kenneth N. McGuinness^1#a^, Weilan Pan^2^, Robert P. Sheridan^1^, Grant Murphy^2^, Alejandro Crespo^1#b*^**

^1^Modeling and Informatics, Merck & Co., Inc., Kenilworth, New Jersey, United States of America

^2^Biochemical Engineering and Structure, Merck & Co., Inc., Rahway, New Jersey, United States of America

**^#a^Current Address: Department of Biochemistry and Microbiology, Rutgers University, New Brunswick, New Jersey, United States America**

**^#b^Current Address: Drug Structure, Prediction, and Design, EMD Serono Research and Development Institute, Inc., Billerica, Massachusetts, United States America**

***Corresponding author**

E-mail: [**alejandro.crespo@emdserono.com**](mailto:alejandro.crespo@emdserono.com) **(AC)**

**Running title: Applicability domain and protein thermostability**

Table A. Statistics of dTm QSPR model predictions.

| Type | | Model | Minutes/  Mutant | r^2^ | ρ^2^ | MAE (℃) | MCC | AUC |
| --- | --- | --- | --- | --- | --- | --- | --- | --- |
| Average | | dT_m_ | 0.01 | 0.20 | 0.2 | 3.53 | 0.25 | 0.71 |
| Sequence | | Amino acid (A) | 3*10^-3^ | 0.07 | 0.07 | 3.83 | 0.02 | 0.63 |
| Structure | | Local (L) | 6*10^-3^ | 0.32 | 0.33 | 3.18 | 0.34 | 0.74 |
|  |  | Global(G) | 0.53 | 0.21 | 0.16 | 3.54 | 0.3 | 0.68 |
| Sequence  Structure | | LG | 0.54 | 0.36 | 0.33 | 3.13 | 0.34 | 0.73 |
|  |  | AL | 9*10^-3^ | 0.33 | 0.31 | 3.18 | 0.27 | 0.71 |
|  |  | AG | 0.54 | 0.24 | 0.2 | 3.45 | 0.31 | 0.71 |
|  |  | ALG | 0.54 | 0.36 | 0.32 | 3.09 | 0.36 | 0.72 |
| Energy, sequence and structure | BL | totE | 0.50 | 0.16 | 0.15 | 3.62 | 0.11 | 0.67 |
|  |  | indE |  | 0.29 | 0.26 | 3.31 | 0.21 | 0.71 |
|  |  | tiE |  | 0.32 | 0.32 | 3.18 | 0.23 | 0.73 |
|  |  | tiE_ALG | 1.04 | 0.41 | 0.39 | 2.95 | 0.36 | 0.76 |
|  | DS | totE | 0.80 | 0.14 | 0.14 | 3.63 | 0.12 | 0.66 |
|  |  | indE |  | 0.20 | 0.19 | 3.49 | 0.18 | 0.68 |
|  |  | tiE |  | 0.21 | 0.20 | 3.49 | 0.2 | 0.68 |
|  |  | tiE_ALG |  | 0.39 | 0.38 | 2.97 | 0.33 | 0.74 |
|  | Cart | totE | 20 | 0.26 | 0.24 | 3.40 | 0.11 | 0.7 |
|  |  | indE |  | 0.32 | 0.32 | 3.20 | 0.26 | 0.74 |
|  |  | tiE |  | 0.39 | 0.38 | 3.00 | 0.31 | 0.77 |
|  |  | tiE_ALG |  | 0.46 | 0.44 | 2.79 | 0.36 | 0.79 |
|  | MOE | totE | 0.53 | 0.09 | 0.08 | 3.80 | -0.02 | 0.59 |
|  |  | indE |  | 0.35 | 0.34 | 3.11 | 0.37 | 0.74 |
|  |  | tiE |  | 0.40 | 0.37 | 2.96 | 0.34 | 0.74 |
|  |  | tiE_ALG |  | 0.43 | 0.40 | 2.87 | 0.41 | 0.75 |
|  | Mono | totE | 101 | 0.15 | 0.15 | 3.69 | 0.08 | 0.67 |
|  |  | indE |  | 0.29 | 0.26 | 3.31 | 0.27 | 0.72 |
|  |  | tiE |  | 0.33 | 0.31 | 3.19 | 0.31 | 0.75 |
|  |  | tiE_ALG |  | 0.43 | 0.40 | 2.88 | 0.34 | 0.77 |
|  | All | All | 129 | 0.51 | 0.48 | 2.67 | 0.4 | 0.8 |
